# Electrochemically Active Sensing Materials and Multi-Material Joints in Aerospace Corrosion Health Monitoring: Transduction, Representativeness and Validation Requirements

**DOI:** 10.3390/ma19173653

**Published:** 2026-08-27

**Authors:** Patryk Ciężak, Andrzej Leski, Krzysztof Dragan, Piotr Synaszko, Michał Sałaciński

**Affiliations:** 1 Faculty of Mechatronics, Military University of Technology, ul. gen. Sylwestra Kaliskiego 2, 00-908 Warsaw, Poland; 2 Łukasiewicz Research Network—Institute of Aviation, al. Krakowska 110/114, 02-256 Warsaw, Poland; 3 Air Force Institute of Technology, ul. Księcia Bolesława 6, 01-494 Warsaw, Poland

**Keywords:** corrosion health monitoring, aerospace aluminium alloys, electrochemical sensors, electrodeposition, conducting polymers, MXenes, atmospheric corrosion, galvanic corrosion, multi-material joints, nondestructive testing

## Abstract

Corrosion of airframe alloys is managed by scheduled inspections rather than measurement. Continuous monitoring could change this. Progress in electrochemically active sensing materials has increased laboratory detectability, yet little has reached operational aircraft. We argue that the limiting factor is not sensitivity but representativeness: whether a sensor’s response reflects the true condition of the structure it monitors. We treat aerospace corrosion as a six-stage cascade and map each material and transduction principle onto the stage it observes. We review the electrochemical processing routes that set electrodes’ morphology and stability and show that the processing parameters strongly affect reported reproducibility when the process’s control is left unstated. We propose an engineering-relevant framework spanning material, environmental, and electrochemical representativeness, plus decision relevance. Across the reviewed corpus, the highest analytical sensitivity tends to coincide with the lowest material representativeness, an apparent qualitative trade-off rather than a demonstrated statistical relationship. No identified system closes the chain from the signal to a damage-based maintenance decision without independent nondestructive verification. Of the performance figures that could be traced to primary studies, none was obtained on an aerospace alloy under airframe-representative exposure, the field’s principal gap. At multi-material joints, the problem inverts: carbon-fibre composites drive alloy’s dissolution while its own matrix degrades, so surface-treatment processing, rather than sensor choice, sets the outcome. We conclude with a staged evidence architecture that couples continuous sensing to eddy current, ultrasonic, and thermographic inspection.

## 1. Introduction

Corrosion is a maintenance problem before it becomes a safety problem. In transport aircraft, it concentrates in lap joints, around fasteners, and beneath coatings, in wheel wells, drainage paths, cargo floors, and avionics bays—locations that are enclosed, that retain electrolyte, and that cannot be inspected without disassembly. The result is an inspection regime driven by calendar and flight-hour intervals rather than by the state of the structure, with the cost of removing and reinstalling the structure to examine metal that usually turns out to be sound.

The materials that make this problem acute are well characterised. In AA2024-T3, attack begins within minutes of chloride exposure at intermetallic particles: S-phase (Al_2_CuMg) dealloys first [1], trenching follows at Al-Cu-Fe-Mn and Al-Cu-Fe-Mn-Si particles, and clusters of particles then corrode co-operatively, producing rings of corrosion product several hundred micrometres across [2,3]. Stable pits develop through extensive grain boundary attack followed by grain etch-out rather than by uniform deepening [4,5]. The Volta potential differences that drive this were mapped by scanning Kelvin probe force microscopy three decades ago [6], and the role of Cu-enriched precipitates in pitting susceptibility was established earlier still [7,8]. Dealloying of constituent particles and dispersoids remains the accepted initiation mechanism in aerospace aluminium [9], and electron tomography has resolved the subsurface morphology [10]. AA7075-T6 degrades by a different route to the same end. Attack initiates at Fe-bearing constituent particles (principally Al_7_Cu_2_Fe and (Al,Cu)_6_(Fe,Cu), which are cathodic with respect to the matrix [11,12,13]), but propagation is governed by MgZn_2_ precipitates decorating the grain boundaries: intergranular penetration develops first, then converts to exfoliation as corrosion product wedges the grain structure apart, and the same boundary chemistry controls susceptibility to stress corrosion cracking [14,15,16]. Retrogression and re-ageing tempers alter the boundary precipitates’ size, spacing, and continuity, and corrosion resistance tracks those morphological parameters rather than bulk composition [14,15]. What matters for monitoring is the consequence, and it is the same for both alloys: structural integrity is governed by localised, subsurface, morphologically irregular attacks whose severity depends on grain boundary and particle microstructure—not by an area-averaged corrosion rate.

Continuous monitoring is a natural alternative to interval-based inspection, and the sensing materials community has supplied an extensive set of candidates. Graphene and its derivatives, carbon nanotubes (CNTs), two-dimensional transition metal carbides and nitrides (MXenes), conducting polymers such as polyaniline (PANI), polypyrrole (PPy) and poly(3,4-ethylenedioxythiophene) (PEDOT), MOFs, nanostructured metal oxides, and printed composite electrodes have all been demonstrated to be corrosion-relevant sensing layers [17,18,19,20,21,22,23]. Reviews of these classes are numerous and of high quality [18,19,20,24,25], and parallel surveys exist for corrosion sensing in pipeline and process infrastructure, where deployment experience is longer than in aerospace [26]. Reviews addressing aerospace corrosion monitoring specifically are rare; Li et al. [17] remains the closest, and it is organised around graphene rather than around the airframe.

The corrosion monitoring literature has developed largely in parallel. Atmospheric corrosion monitoring has an established toolkit (electrochemical noise (EN), electrochemical impedance spectroscopy (EIS), linear polarisation resistance (LPR), electrical resistance (ER) probes, and atmospheric corrosion monitor (ACM) galvanic sensors) whose principles and thin electrolyte limitations have been reviewed thoroughly [27,28,29]. Several techniques have field-validated data behind them. ACM sensors have been correlated with components’ durability in automotive service [30]; ER probes have been automated and validated in controlled atmospheres [31] and under multi-droplet wetting [32]; commercial ER, LPR, and ultrasonic thickness devices have been compared directly on the same specimens [33]. Mansfeld et al. established instantaneous atmospheric corrosion rate measurements 40 years ago [34]. Tan set out both the diagnostic value of EN for localised attack [35] and the specific difficulties of measuring corrosion in highly resistive and inhomogeneous media [36]—the regime that applies inside an aircraft compartment.

These two strands of the literature rarely meet. Sensing materials papers report detection limits and response times benchmarked against analyte concentration; corrosion monitoring papers report the corrosion current density, charge, and thickness loss against mass-loss coupons. Neither routinely reports whether the sensing element degrades like the alloy it represents, whether it experiences the same microenvironment, or what a maintenance organisation should do when the signal changes. Where machine learning has been applied successfully to corrosion sensor data [37], the prediction target was the sensor’s own future signal rather than verified structural damage.

That gap defines this review. We do not attempt another survey of sensing chemistry. We ask a narrower and more consequential question: under what conditions does the response of an electrochemically active sensing material constitute engineering evidence about an aerospace structure? Answering it requires three things usually treated separately—an explicit model of which stage of corrosion each sensor observes, an account of how electrochemical processing determines the reproducibility of the sensing electrode, and a criterion for converting a signal into a maintenance decision. Because the corpus assembled to answer it spans very different degrees of directness to an aerospace structure, from sensors tested on aerospace alloys to biosensing studies included only for transduction mechanisms, Section 2.1 introduces a four-level evidence hierarchy (D1–D4) that is used to qualify every subsequent claim in this review.

Section 2 states the literature selection strategy. Section 3 develops the corrosion cascade used as the reference frame throughout. Section 4 reviews the sensing material classes and the electrochemical processing routes used to fabricate them. Section 5 maps the transduction mechanisms onto the cascade stages. Section 6 introduces the four-dimensional representativeness framework and applies it. Section 7 sets out the coupling between continuous sensing and nondestructive testing (NDT). Section 8 identifies the specific experiments that are missing, and Section 9 states the conclusions.

## 2. Scope, Evidence Base, and Its Limits

This paper is a critical narrative review, not a systematic one. We state this explicitly because the distinction governs how its conclusions should be read: the corpus was assembled to support an argument about representativeness, not to establish the complete state of a defined branch of the literature, and no claim is made that it is exhaustive.

Sources were retrieved from Scopus, Web of Science Core Collection, and IEEE Xplore for the period 1986–2026, the lower bound set by the first quantitative atmospheric corrosion rate monitor [34], and supplemented by reference list tracing from the reviews and primary studies identified. Search terms combined three groups: (i) *corrosion monitoring*, *corrosion sensor*, *and corrosion health monitoring*; (ii) *aerospace*, *aircraft*, *airframe*, *aluminium alloy*, *AA2024*, *and AA7075*; (iii) material and method descriptors (*graphene*, *MXene*, *conducting polymer*, *metal–organic framework*, *printed electrode*, *electrodeposition*, *anodising*, *EIS*, *electrochemical noise*, *electrical resistance probe*, *and fibre-optic*). Sources were retained where they reported a quantified response to a corrosion-relevant stimulus, the composition and fabrication route of the sensing element, or exposure conditions and independent verification. Purely biomedical and gas-phase analyte studies were excluded unless the transduction mechanism transfers directly to corrosion-relevant species. Because this is a narrative rather than a systematic review, exhaustive record counts were not the objective; retained and excluded sources were judged individually against the criteria above.

### 2.1. Hierarchy of Evidence

The corpus spans very different degrees of directness, from sensors tested on aerospace alloys to biosensing studies included only for their transduction mechanism. Treating these as equivalent would be misleading, so each claim in this review should be read against the following hierarchy. Assignment to a level required the following, checked against each source: D1 required (i) the sensing element or measurement to be on a specimen of an aerospace-qualified alloy (e.g., AA2024, AA7075, Ti-6Al-4V) or on an airframe structure, (ii) exposure conditions reproducing or closely approximating an airframe-representative environment, and (iii) a reported corrosion measurement rather than a mechanistic or protection-only result. D2 required Condition (iii) together with a transferable corrosion mechanism demonstrated on a non-aerospace alloy, geometry, or environment. D3 required a quantified response to a corrosion-relevant species or condition without a structural alloy present. D4 comprised processing or mechanistic studies that constrain sensing electrode design without themselves reporting a sensing response.

- **D1—Direct aerospace corrosion monitoring evidence:** a quantified corrosion measurement obtained on an aerospace alloy or airframe structure under corrosion-relevant exposure. As the quantitative synthesis in Section 5 shows, no primary study in this corpus meets D1 in full; the closest are airframe-specific sensor developments [38] and installed-NDT demonstrations on aircraft structures [39], neither of which reports a validated continuous corrosion measurement on the alloy. Review [17] is secondary and is used only for orientation.

Applying these criteria to the 103 references cited in this review gives the following distribution: 0 references meet D1 (0%); 18 meet D2 (17.5%), drawn from the atmospheric, automotive, pipeline, and marine corrosion monitoring literature [30,31,32,33,34,37,40,41,42,43,44,45,46,47,48,49,50,51]; 17 meet D3 (16.5%), drawn from the sensing materials literature [18,19,20,21,22,23,24,25,52,53,54,55,56,57,58,59,60]; and 38 meet D4 (36.9%), drawn from processing, anodising, and alloy-metallurgy studies [1,2,3,4,5,6,7,8,9,10,11,12,13,16,61,62,63,64,65,66,67,68,69,70,71,72,73,74,75,76,77,78,79,80,81,82,83,84]. The remaining 30 references (29.1%) are reviews, background metallurgy, NDT method development or aircraft economics, and certification sources cited for orientation rather than as part of the sensing evidence corpus and are not assigned a D-level. Three distinct forms of validation also appear across this corpus and are frequently conflated. Sensor validation establishes that the sensing element responds reproducibly to a controlled stimulus under defined conditions, for example, calibration against a known chloride concentration or humidity. Validation of corrosion prediction establishes that the sensors’ output correlates with an independently measured corrosion outcome, most often mass loss or thickness loss on a coupon exposed to the same environment [30,33,41]. Structural damage validation establishes that the sensors’ output, or the corrosion state inferred from it, corresponds to a specific, independently confirmed form of structural degradation: pit morphology, crack initiation, or NDT-detected material loss at a defined location. Only the last constitutes engineering evidence in the sense argued in Section 6. Where this review refers to a study as validated without further qualification, the intended level should be read from the accompanying D1–D4 classification and the reported validation quality, and not assumed to be structural.

- **D2—Transferable corrosion monitoring evidence:** atmospheric, automotive, pipeline, or marine monitoring where the measurand and degradation mechanism transfer but the alloy, geometry, or environment differ [30,31,32,33,34,37,40,41,42,43,44,45,46,47,48,49,50,51]. Most of the quantitative performance reviewed here sits at this level.
- **D3—Corrosion-relevant sensing-material evidence:** materials characterised against corrosion-relevant species or conditions but not against a structural alloy [18,19,20,21,22,23,24,25,52,53,54,55,56,57,58,59,60].
- **D4—Processing or mechanistic evidence:** coating, anodising, electrodeposition and alloy-metallurgy studies that constrain what a sensing electrode can be without themselves being sensing studies [1,2,3,4,5,6,7,8,9,10,11,12,13,16,61,62,63,64,65,66,67,68,69,70,71,72,73,74,75,76,77,78,79,80,81,82,83,84]. Eddy current and thermographic inspection studies [39,85] provide direct aerospace evidence of damage detection but not of continuous corrosion sensing, and are treated as such.

Two axes are therefore in play and are kept separate throughout: application directness (D1–D4 above) and, independently, validation quality (independent field/component validation, coupon comparison, NDT comparison, no independent validation, or review or mechanistic only). Statements resting only on D3 or D4, or on weak validation, are identified where the distinction affects the argument. The framework of Section 6 is a synthesis across all levels, offered as a structured hypothesis rather than an established model.

### 2.2. Limitations of the Evidence Base

Two limitations constrain everything that follows. Few studies report sensing materials’ performance under simultaneous cyclic humidity, chloride deposition, and temperature variation, and fewer still over durations comparable with a scheduled maintenance interval. Direct comparison across studies is generally not possible, because the exposure protocols, electrolyte compositions, electrode geometries and reported metrics differ from paper to paper [27,29]. Comparative statements between material classes are therefore qualitative throughout and are identified as such.

### 2.3. Use of Generative Artificial Intelligence in Manuscript Preparation

OpenAI ChatGPT (GPT-5.6 Thinking; OpenAI, San Francisco, CA, USA; accessed 26 July 2026) was used during manuscript preparation for English language editing, stylistic refinement, consistency checking, and the preparation and refinement of schematic figures. It was not used to select literature autonomously, generate or modify primary data, perform statistical analyses, assign evidence levels, or formulate the scientific conclusions. Every AI-assisted output was checked against the cited sources and revised by the authors, who take full responsibility for the final content.

## 3. Corrosion as a Cascade: Defining What Is Being Measured

Corrosion is not a single measurable quantity. It is a sequence of physically distinct processes and a sensor intercepts that sequence at one point. The six-stage division introduced below is the authors’ organising synthesis, developed to reconcile the sensing materials literature and the corrosion monitoring literature that report against different measurands (Section 1); the physical description of each stage, and the transitions between them, rests on established mechanistic literature cited individually stage-by-stage below and summarised with the sources in Table 1. This is the organising principle of the review, because most disagreements in the literature about sensors’ “accuracy” dissolve once it is clear that two sensors were observing different stages.

Six stages are useful for aerospace structures.

1. **Environmental exposure**—humidity, temperature, chloride, and sulphur deposition. Necessary but not sufficient.
2. **Electrolyte formation**—condensation, deliquescence, or trapped water producing a continuous conductive film; below a critical thickness and continuity, electrochemistry does not proceed [36].
3. **Electrochemical activation**—coupled anodic and cathodic reactions established, passive film broken down, and a measurable corrosion current.
4. **Localised propagation**—pitting, intergranular attack, exfoliation, crevices, and galvanic attack at interfaces. This governs structural integrity in AA2024-T3 and AA7075-T6 [4,8,9].
5. **Cumulative material loss**—integrated section loss, measurable as a change in thickness or resistance.
6. **Structural damage**—loss of load-carrying capability, fastener hole degradation, disbonding, and crack initiation.

**Figure 1:**
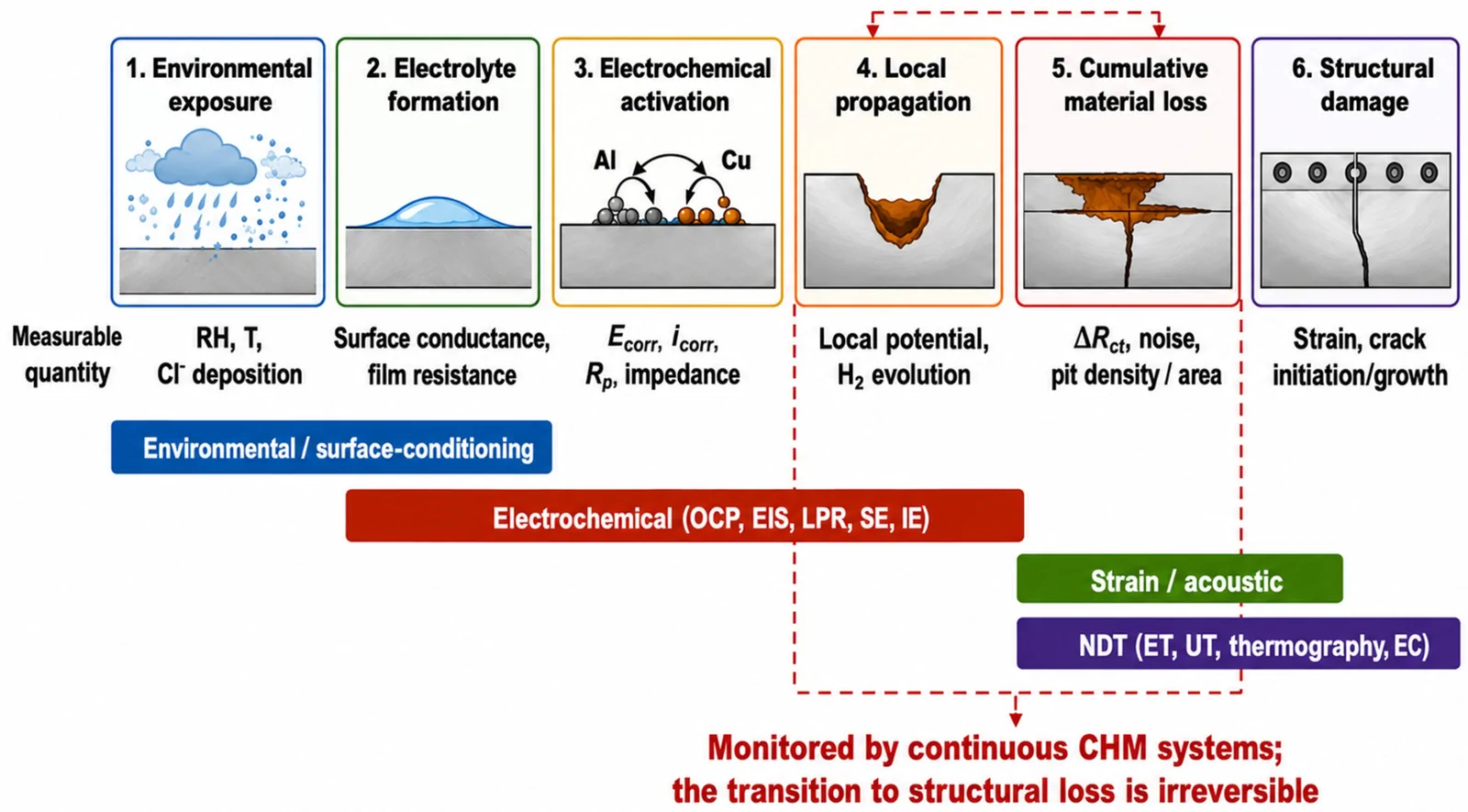
Six-stage corrosion cascade in aerospace structures and the stages accessible to each class of monitoring technique. The measurable quantity associated with each stage is given beneath the stage. The dashed region marks the transition from localised propagation to measurable section loss, where no continuous technique reviewed here provides unambiguous evidence.

The stages are a heuristic decision framework rather than a mechanistic model: in a real joint, localised propagation, cumulative loss, and structural damage overlap in time, and the boundaries between them are diffuse. What the framework asserts is narrower and more useful—that each technique has a primary evidence stage, the stage about which its signal is least ambiguous (Figure 1, Table 1). A technique may be sensitive to adjacent stages without being diagnostic for them. A humidity and surface conductance array reports Stages 1–2 reliably and says nothing about whether metal is dissolving. An ACM galvanic sensor reports Stage 3 as a charge integral that must be converted to depth through a calibration that is itself environment-dependent [41,42]. An ER probe reports Stage 5 directly and cumulatively but averages over the sensing track and therefore under-represents localised morphology [31,32,33]. EN reports transients associated with Stage 4 [35], but its interpretation depends heavily on the signal processing applied. Eddy current and ultrasonic inspection report Stages 5–6 with spatial resolution, but only when the inspection is performed.

**Table 1:** Corrosion cascade stages, the physical processes involved, the quantities that can be measured, the techniques that observe each stage, and what those techniques cannot resolve.

Stage	Physical Process	Measurable Quantity	Techniques with This as the Primary Evidence Stage	Not Resolved Unambiguously	Refs.
1. Environmental exposure	RH, T, Cl^−^, and S deposition, pollutant loading	RH (%), T (°C), deposition rate (mg m^−2^ d^−1^)	RH/T sensors, deposition coupons, ACM (partially)	Whether an electrolyte formed; whether metal dissolved	[27,42]
2. Electrolyte formation	Condensation, deliquescence, retained water; film continuity	Surface conductance (S), time of wetness (h), galvanic current onset	Surface conductance arrays, ACM, quartz crystal microbalance (QCM)	Whether the film supports sustained electrochemistry	[27,32,34,36,40]
3. Electrochemical activation	Coupled anodic/cathodic reactions; passive film breakdown	i_corr (A cm^−2^), charge (C), R_p (Ω cm^2^), impedance spectrum	ACM/galvanic, ZRA, LPR, EIS	Location and morphology of attack; conversion to depth is model-dependent	[28,30,34,40,41]
4. Localised propagation	Pitting, IGC, exfoliation, crevices, and galvanic attack	Current/potential transients; transient rate and amplitude	EN	Deterministic damage size; interpretation is process-dependent	[4,9,35,36]
5. Cumulative material loss	Integrated section loss	ΔR/R_0_ (–), thickness loss (µm)	ER probes, UT thickness, mass loss coupons	Whether loss is uniform or localised; when it occurred	[31,32,33]
6. Structural damage	Load path degradation, disbonding, crack initiation	Defect size (mm), remaining thickness (mm)	ET, PEC, PEC thermography, UT	Ongoing activity; requires an inspection event	[33,39,85]

Two consequences recur throughout this review. A sensor cannot be validated against a quantity it does not measure—comparing an environmental sensor against mass loss coupons tests the environment-to-corrosion model, not the sensor. Moreover, a system covering only the early stages cannot support a damage tolerance decision, however sensitive, because it provides no evidence at Stage 5 or 6 (Section 7).

## 4. Sensing Materials and Their Electrochemical Processing

### 4.1. Requirements Specific to Airframe Deployment

The operating envelope for an airframe corrosion sensor differs from the laboratory in ways that eliminate many otherwise excellent materials. The sensing element must survive repeated wet–dry cycling, chloride and hydraulic fluid contamination, temperature excursions from roughly −55 °C at altitude to well above ambient on a hot ramp, vibration, and service intervals measured in years. It must add negligible mass, conform to a curved and confined geometry, and it must not itself become a corrosion initiation site—an installed sensor that creates a crevice or a galvanic couple with the structure has made the problem worse.

These constraints reorder the usual priorities: baseline stability over years above the detection limit, reversibility and hysteresis above peak sensitivity, and manufacturing reproducibility above both. A distributed network of nominally identical sensors is of little use if their responses are not comparable. Table 2 summarises the six material classes reviewed in Section 4.2, Section 4.3, Section 4.4, Section 4.5 and Section 4.6 against these requirements.

A further constraint is often overlooked. Aerospace aluminium is not bare metal. It carries a qualified protection system (an anodic oxide, an inhibited primer, and a topcoat [61,62]), and any sensor must be compatible with that system or must be integrated into it. Chromate conversion coatings and chromic acid anodising, whose inhibition mechanism was established decades ago [72], are being displaced under the EU’s Registration, Evaluation, Authorisation and Restriction of Chemicals regulation (REACH), and the replacement chemistries (tartaric–sulphuric anodising, trivalent chromium process, Li and Ce salts, hybrid sol–gel layers) are still being qualified [61,62,63,64,65,66]. This transition is an opportunity: a protection system under redesign is a protection system into which sensing functionality can be designed. Buchheit et al. recognised this two decades ago, treating active corrosion protection and corrosion sensing as one problem in chromate-free organic coatings [64].

### 4.2. Carbon-Based Nanomaterials

Graphene, graphene oxide (GO), reduced graphene oxide (rGO), CNTs, and laser-induced graphene are the most extensively studied sensing materials in this field, with electrical conductivity, carrier mobility, and specific surface area far beyond conventional electrode materials [17,21,22]. Oxygen-containing groups on GO and rGO provide functionalisation sites for selective interaction with chloride, dissolved metal ions, and corrosion products, and CNT networks provide percolating conductivity that survives the strain associated with flexible substrates [17,21].

The relevant weakness for aerospace is drift rather than performance: the response depends on humidity, contamination, ultraviolet exposure, and slow ageing of the surface functional groups [17,22]. Moreover, a graphene electrode does not corrode like AA2024-T3, so any inference about the alloy passes through a correlation that must be re-established for each configuration. Their primary evidence stages are 1–3.

### 4.3. MXenes

MXenes combine metallic conductivity with hydrophilic, chemically active surface terminations (–O, –OH, –F), and a layered structure permitting fast ion transport [23,52]. Terminations can be tuned to alter selectivity toward corrosion-relevant species, and MXene inks integrate readily into printed and flexible devices [19,23]. Their reported sensitivity is among the highest of any class reviewed here under laboratory single-analyte conditions, though a direct cross-class comparison under matched conditions is not available in this corpus [52,53].

The obstacle is oxidative stability: MXene flakes degrade in humid oxygenated environments, precisely the condition a corrosion sensor observes, with reported lifetimes short relative to the maintenance intervals [23,52,53]. Hybridisation with MOFs and encapsulation have been proposed [54], but no long-term data under cyclic wetting have been identified. Until such a dataset exists, MXenes remain a laboratory material here.

### 4.4. Conducting Polymers

PANI, PPy, and PEDOT change conductivity with the oxidation state, which couples them directly to the electrochemical potential of the interface they contact [20,55]. They are mechanically compliant, they deposit from solution, and they can be loaded with corrosion inhibitors to combine sensing with protection. Polyaniline-reinforced epoxy adhesive has been used to suppress galvanic corrosion in bond-riveted AA5083/CFRP joints, the clearest existing demonstration of the dual function in a multi-material assembly [77]—the dual function anticipated by Buchheit et al. [64] and now actively pursued in chromate-free coating development [61,65]. Their mixed ionic–electronic conduction makes them responsive to both electrolyte formation and electrochemical activation, spanning Stages 2–3.

Their limitation is measurement stability: hysteresis, baseline drift, swelling under repeated wetting, and chemical ageing progressively alter their conductivity [20,55]. Film morphology and electrode geometry influence the response as much as the polymer chemistry, which is why nominally identical formulations from different laboratories are hard to compare—a processing question, treated in Section 4.7.

### 4.5. Metal–Organic Frameworks and Nanostructured Metal Oxides

MOFs offer high specific surface area and tailorable pore chemistry, giving them, on the basis of the pore-tuning strategy alone, the potential for the highest chemical selectivity of any class reviewed here for specific ionic species. As the following paragraph notes, this potential has not been demonstrated against structural alloy corrosion and remains an inference from the biosensing and aqueous analyte literature [24,25,58,59]. Nanostructured ZnO, TiO_2_, SnO_2_, and Fe_2_O_3_ provide chemically active surfaces that are responsive to oxygen, humidity, pH, and dissolved metal ions and perform well on conductive supports [25,57].

An important caveat applies: the MOF sensing literature is directed overwhelmingly at aqueous analyte detection and biosensing [24,25,56,57,58,59], and no direct validation for structural alloy corrosion monitoring was identified. This is D3 evidence in the hierarchy of Section 2.1. Hydrolytic stability, response to contamination, and baseline stability under cyclic humidity are the specific gaps.

### 4.6. Printed Composite Electrodes and Flexible Platforms

Screen, inkjet, and aerosol jet printing allow functional inks containing graphene, CNTs, MXenes, metallic nanoparticles, or conducting polymers to be deposited onto flexible substrates or directly onto structural components [19,22]. The engineering advantages are decisive: low mass, conformability, scalable array production, and access to confined compartments that conventional probes cannot reach.

The corresponding weakness is reproducibility: inks’ rheology, deposition, sintering and encapsulation produce measurable differences between nominally identical sensors [19]. For one laboratory device, this is a nuisance; for a distributed network compared across an airframe, it is disqualifying unless the process control is quantified. Sensor-to-sensor variation is rarely stated in this branch of the literature (Section 8).

### 4.7. Electrochemical Processing Routes for Sensing Electrode Fabrication

**Figure 2:**
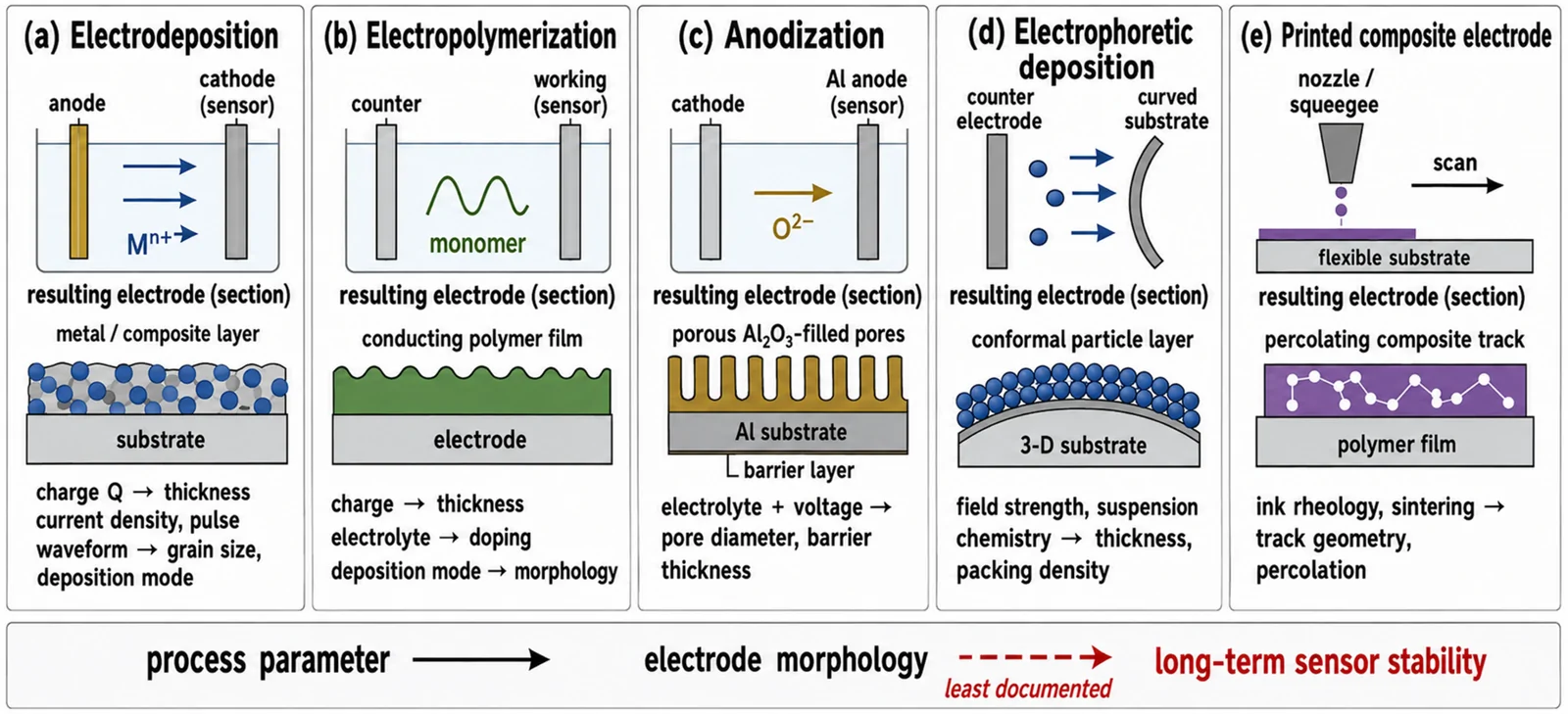
Electrochemical and printing routes for the fabrication of corrosion-sensing electrodes: (**a**) electrodeposition; (**b**) electropolymerisation; (**c**) anodic oxidation; (**d**) electrophoretic deposition; (**e**) printed composite electrodes. For each route, the process’s arrangement (upper sub-panel), the resulting electrode section (lower sub-panel), and the parameters controlling the morphology are indicated. The bar beneath the panels identifies the chain from processing parameter to long-term sensor stability; the final link is the least documented in the reviewed corpus.

The reproducibility problem above is strongly process-dependent. Processing is a major and experimentally tractable determinant of reproducibility and long-term stability, alongside material selection. The routes are summarised in Table 3 and shown schematically in Figure 2; the points below are what the table cannot carry.

Electrodeposition controls thickness through the charge and morphology through the current density, bath composition, and pulse waveform. Parametric sensitivity is well quantified in the coatings literature: pulse frequency and the duty cycle strongly influence the nucleation rate and therefore grain size, with pulsed deposition yielding finer, denser structures and higher particle incorporation than direct current at equal average current densities [68,69]. The co-deposited particle fraction is set by bath loading and agitation, and local current density variation across the substrate is the principal source of non-uniform particle distribution [68]—that is, of electrode-to-electrode variability. For sensing corrosion, the route can, in principle, produce an electrode of a representative alloy composition, but composition is not microstructure, and an electrodeposited layer will not reproduce the temper-dependent intermetallic distribution, grain structure, and grain boundary precipitation that govern attack in T3 or T6 materials (Section 6.1). It is therefore better described as compositionally representative or electrochemically calibrated than as metallurgically representative—the latter is more nearly achieved by a coupon or thin foil of the actual alloy. Electrochemical deposition has been used to form cerium conversion layers on anodised AA2024-T3, showing compatibility with an already anodised airframe surface [61,62], and electrodeposited layered double hydroxide films offer a further route to functional, ion-exchanging layers on aluminium [82].

**Electropolymerisation** deposits PANI, PPy, and PEDOT directly, with the thickness set by the charge, doping by the supporting electrolyte, and morphology by the deposition mode [20,55,73]. Because film morphology governs the drift and hysteresis described in Section 4.4, the deposition protocol is the primary determinant of long-term stability—a point that is under-reported, since the deposition parameters are commonly given but rarely alongside multi-month stability data.

Anodic oxidation produces porous oxide, serving simultaneously as a sensor substrate and for dielectric isolation and corrosion protection. Pore geometry, barrier thickness, and adhesion are controlled through the electrolyte’s chemistry and voltage; the resulting barrier can be quantified by impedance spectroscopy [84]; and anodising measurably changes the galvanic behaviour of a coupled assembly [78]. The aerospace-qualified variants (phosphoric, phosphoric–sulphuric, sulphuric, and tartaric–sulphuric) are well characterised, each optimised for a different balance of adhesion, protection, and fatigue performance [62,70,71]. Among the routes compared in Table 3, anodic oxidation offers a distinctive combination of high aerospace process maturity and a porous layer that can host electroactive species without requiring an additional deposition step; on this specific basis, rather than on demonstrated sensing performance, we consider it the most readily integrable route available. Note, however, that filling the pores or embedding electrodes modifies the layer and would require re-qualification against fatigue, adhesion, bonding, and galvanic compatibility. That, as Table 3 shows, it is also the route with no direct sensing demonstration identified in this corpus, so its practical performance as a sensor substrate remains untested relative to routes with lower process maturity but existing laboratory demonstrations.

Electrophoretic deposition provides a conformal coating of complex geometry from particle suspensions, which is relevant for graphene, MXene, or MOF layers on non-planar components where printing is impractical. Conversion and sol–gel layers, applicable by dipping, spraying, or electrodeposition from the sol [81,83], provide the encapsulation that determines whether the sensor experiences the same microenvironment as the structure (Section 6.2). Hybrid sol–gel systems developed as chromate replacements [61,63,66,74] apply directly. Encapsulation is usually described as a packaging detail; we argue it is a primary design variable, because it defines the sensor’s transfer function to the environment.

A consistent pattern emerges: each route offers a controlled, scalable path from bath or ink to a functional electrode, and for each, the influence of the processing parameters on long-term stability is under-characterised relative to their influence on the initial sensitivity. This is the most tractable gap identified here, because closing it requires no new chemistry—only systematic parametric studies.

**Table 2:** Sensing material classes: composition, sensing mechanisms, cascade stages addressed, and the specific limitations for airframe deployment.

Material Class	Typical Composition	Key Sensing Mechanism	Cascade Stages	Principal Limitation for Airframe Use	Refs.
Carbon nanomaterials	Graphene, GO, rGO, CNT, LIG	Charge transfer at functionalised surface; percolation conductivity (threshold ≈1 wt% MWCNT in epoxy; 10^−16^→10^−2^ S cm^−1^)	1–3	Electrochemically unlike Al alloys; drift with humidity, UV, and contamination; raises cathodic area if present in adjacent composite	[17,21,22,86]
MXenes	Ti_3_C_2_T_X_ and related carbides/nitrides	Ion intercalation; termination-dependent charge transfer	2–3	Oxidative degradation in humid oxygenated environments	[23,52,53,54]
Conducting polymers	PANI, PPy, PEDOT	Oxidation-state-dependent conductivity; mixed ionic–electronic conduction	2–3	Hysteresis, swelling, and baseline drift under wet–dry cycling	[20,55,64]
MOFs	Zr-, Cu-, Fe-based frameworks	Selective adsorption in tailored pores	2–3	No direct validation for structural alloy corrosion; hydrolytic stability	[24,25,56,57,58,59]
Nanostructured metal oxides	ZnO, TiO_2_, SnO_2_, Fe_2_O_3_	Surface reaction with O_2_, H_2_O, H^+^, metal ions	1–3	Baseline stability under cyclic humidity and contamination	[25,57]
Printed composites	Functional inks on flexible substrates	Depends on filler; typically resistive or capacitive	1–3, 5	Sensor-to-sensor reproducibility rarely quantified	[19,22]
Representative alloy elements	AA2024, AA7075, Fe/Cu couples	Sacrificial dissolution; galvanic coupling	3, 5	Lower analytical sensitivity; morphology averaging	[30,31,33,40]
CFRP (as adjacent structure, not sensor)	Carbon-fibre/epoxy or BMI	Cathodic O_2_ reduction; peroxide pathway	Drives 3–4 in coupled metal	Not a sensor; sets the corrosion driving force at joints. C_dl 3–16 µF cm^−2^ basal vs. 50–70 µF cm^−2^ edge	[67,86]

**Table 3:** Electrochemical processing routes for corrosion-sensing electrode fabrication. Aerospace process maturity is rated high, medium, low–medium, or low; sensing demonstrations and maintenance interval stability evidence are reported separately. ‘Not identified’ denotes absence from this narrative corpus (Section 2), not absence from the literature.

Route	Materials Deposited	Control Parameter → Morphology	Aerospace Process Maturity	Direct Sensing Demonstration	Sensor Stability Evidence	Refs.
Electrodeposition	Metals, alloys, metal composites	Charge → thickness; current density and pulse waveform → grain size, roughness	High	Partial	Not identified	[61,62,68,69]
Composite electrodeposition	Metal matrix + dispersed particles	Bath particle loading, agitation → volume fraction	Medium	Not identified	Not identified	[68,69]
Electropolymerisation	PANI, PPy, PEDOT	Charge → thickness; electrolyte → doping; deposition mode → morphology	Low	Yes (laboratory sensors)	Not identified	[20,55,73]
Anodic oxidation (PAA, PSA, SAA, TSA)	Porous Al_2_O_3_ substrate/isolation layer	Electrolyte and voltage → pore diameter, barrier thickness; tartaric acid modifies pore morphology and sealing	High	Not identified (protection only)	Protection data only	[61,62,70,87]
Plasma electrolytic oxidation	Crystalline Al_2_O_3_/mullite layer	Process voltage → crystallinity; 700 V gave ~90% galvanic current reduction vs. ~12% for TFSAA	Medium	Not identified	Protection data only	[67,79,80]
Electrophoretic deposition	Graphene, MXene, MOF particulates	Field strength, suspension chemistry → thickness, packing	Low	Not identified	Not identified	—
Sol–gel/conversion encapsulation	Hybrid silica–zirconia, Ce/Li-doped layers	Precursor chemistry, curing → barrier properties	Medium	Not identified	Protection data only	[61,63,65,66,81,83]
Screen/inkjet/aerosol jet printing	Functional composite inks	Ink rheology, deposition and sintering → track geometry, percolation	Low–medium	Yes (laboratory arrays)	Not identified	[19,22]

### 4.8. Multi-Material Joints: What CFRP Coupling Means for Sensor Design

The classes above are treated in the literature as sensing layers applied to a metallic structure. In a modern airframe, that premise is incomplete: the structure is a multi-material assembly, and its interfaces are where corrosion concentrates. Three findings from that literature change how a sensor at such a joint should be designed and read.

The driving force is set by the joint, not the alloy. Carbon-fibre is conductive and adopts a relatively noble open-circuit potential under marine and atmospheric exposure—reported to be in the range of roughly +0.1 to +0.3 V vs. SCE (depending on the fibre type, exposed fibre fraction, resin, and oxygenation) rather than a single thermodynamic electrode potential, so on contact with a structural alloy in an electrolyte, the composite is cathodically polarised and supports oxygen reduction over its whole wetted area [43,67,86]. The effect is selective rather than uniform. Coupling a graphite/polymer composite to 2014 aluminium, titanium, 316 stainless steel, and Monel in seawater left titanium inactive, shifted stainless steel and Monel from non-corroding to crevice and pitting attacks, and left the aluminium corrosion mode unchanged while blistering the composite [47]. Magnesium alloys behave similarly but more severely [76]. For sensing, galvanic risk at a joint depends on which passive film the coupled alloy carries, so a sensor calibrated at one material pairing does not transfer to another even within the same airframe.

Two processes must be distinguished, not one. Cathodic polarisation degrades the composite as well as the metal. Coupling T-300/934 graphite/epoxy to magnesium for 140 days reduced apparent shear strength by about 30%, with localised delamination of an order of 5 mm per year [43]. The damaging species are oxygen-reduction intermediates rather than the final product: both hydroxyl and perhydroxyl ions evolve at the cathode [45], and hydrogen peroxide attacks the secondary amines of cured epoxy directly [46]. High pH alone is insufficient (unpolarised specimens at pH 13 were undamaged [48]), and matrix chemistry sets the threshold, with bismaleimide degrading under high pH alone and toughening making it worse [88]. Anodic polarisation from stray currents damages the composite by a third route at current densities as low as 1 µA cm^−2^ [44]. For sensing, a signal at such a joint superposes two degradation processes with different thresholds, so attributing it to alloy loss alone will mis-state the condition of the joint.

Nanofillers in the adjacent structure work against the sensing case. Adding multi-walled carbon nanotubes shifts an epoxy matrix from insulating, of the order of 10^−16^ S cm^−1^, to 10^−3^–10^−2^ S cm^−1^ above a percolation threshold near 1 wt% [86], enlarging the composite’s effective cathodic area. Reported consequences vary with whether the coupled metal polarises the CFRP into the diffusion-controlled regime—with a roughly doubled corrosion rate for AA7075 against a CNT-modified glass-fibre composite [89], and increased galvanic effect but reduced uniform and localised attack for 304 stainless steel bonded with CNT-modified epoxy adhesive, because the modified adhesive kept electrolyte from the substrate [90]—but the direction of the mechanism is not disputed. For sensing, the nanomaterials proposed in Section 4.2 and Section 4.3 as high-sensitivity layers are, when deployed millimetres away in the structure, materials that raise the corrosion-driving force the sensor is meant to measure.

Mitigation is a processing variable and it moves the electrochemical operating point. Liu et al. measured an AA7075/CFRP couple in 3.5 wt% NaCl by zero-resistance ammetry: the bare couple stabilised at 58.2 µA cm^−2^ and −0.685 V (vs. Ag/AgCl), while plasma electrolytic oxidation at 700 V gave 6.2 µA cm^−2^ at −0.36 V—a reduction by approximately 90% against roughly 12% for a comparable thin-film sulphuric acid anodising layer [67]. Below 500 V, the layer is amorphous and porous; above 600 V, it crystallises to Al_2_O_3_ and mullite, and the galvanic current falls by roughly an order of magnitude [11,67]. The protection is a barrier effect: the dense crystalline oxide isolates the alloy from the electrolyte and raises the resistance of the couple. It should not be attributed to the accompanying shift in mixed potential: a nobler potential above the pitting potential would, on an electrochemically accessible surface, favour rather than suppress stable pitting, so the benefit follows from the barrier, not from the potential change. The layer thickness was 10–11 µm either way. Pulse waveform is a further lever [79], and design guidelines exist for insulation layers at such joints [75]. For sensing, the treatment reduces corrosion and also relocates the joint’s operating potential and interfacial resistance, so a sensor’s baseline and detection threshold must be set for the treated condition, not the bare one, and re-set if the treatment is repaired in service.

Finally, the carbon surface’s own electrochemical activity is morphology-controlled: double-layer capacitance runs at 3–16 µF cm^−2^ on basal planes against 50–70 µF cm^−2^ on edge sites [86]. Cathodic behaviour is set by the processing history rather than the nominal composition—the conclusion reached for sensing electrodes in Section 4.7, arrived at from the opposite direction.

A further consideration follows directly from this section. The nanomaterials proposed as sensing layers in Section 4.2 and Section 4.3 are, when present in adjacent composite structures rather than isolated as a sensor, exactly the materials shown above to enlarge cathodic area and accelerate galvanic attack on coupled aluminium alloys [86,89,90]. An installed graphene, CNT, or MXene sensor that is electrically continuous with the structure it monitors is therefore not electrochemically neutral: if it forms a low-resistance path to the alloy, it can act as a local cathode and increase, rather than only report, the corrosion it is meant to detect. This risk is distinct from the material representativeness question of Section 6.1, which concerns whether the sensor’s response tracks the alloy’s degradation; here, the concern is that the sensor’s presence changes that degradation. The mitigation follows the same principle applied to CFRP joints above: electrical isolation between the conductive sensing layer and the structure wherever contact is not the deliberate measurement pathway, following the insulation layer approach evaluated for composite/alloy joints by Srinivasan et al. [75]. No study in this corpus reports testing a nanomaterial-based sensor against this failure mode.

### 4.9. Synthesis: No Single Material Satisfies the Requirement Set

Across the classes reviewed, sensitivity, selectivity, environmental durability, material representativeness, and integrability do not co-occur. Carbon nanomaterials and MXenes deliver sensitivity at the cost of representativeness and, for MXenes, stability. MOFs deliver selectivity without demonstrated corrosion relevance. Conducting polymers deliver integrability and dual functionality at the cost of drift. Electrodes fabricated from representative alloys deliver representativeness at the cost of sensitivity. Printed composites deliver integration at the cost of reproducibility. The evidential consequence is explicit: the traceable quantitative performance figures in this field come almost entirely from atmospheric, pipeline, and marine studies, and none has been obtained on an aerospace alloy under airframe-representative exposure.

The reviewed evidence suggests this is a plausible design trade-off, read qualitatively across the six sensing-material classes of Table 2 rather than established through a quantitative correlation, and rather than a temporary state of the art awaiting a better material. The properties that make a material an excellent transducer (high surface area, high charge-transfer efficiency, and tailored surface chemistry) are the same properties that make it electrochemically unlike a structural aluminium alloy. Recognising this explicitly, rather than treating it as a gap in materials development, is a prerequisite for designing monitoring systems that work. Hybrid architectures pairing a representative metallic element with a high-sensitivity transducer are the practical response, as developed in Section 6.

## 5. Transduction Mechanisms and Their Primary Evidence Stages

The sensing material determines what can be detected; the transduction mechanism determines what is reported. Each mechanism is examined here with one question: which cascade stage is its primary evidence stage, and with what confidence? Table 4 summarises the techniques; Table 5 collects the quantitative performance that could be traced to primary sources. This section gives only what those tables cannot.

### 5.1. Electrochemical Transduction

Corrosion is an electrochemical process, which makes electrochemical transduction natural and also creates its characteristic difficulty—the measurement perturbs, or is perturbed by, the interface it observes.

Galvanic and ACM sensors generate current from the potential difference between dissimilar electrodes, typically Fe/Cu or a representative alloy, against a noble counter-electrode [34,40]. Current flows only when a continuous electrolyte film bridges them, making the sensor an unusually direct indicator of Stage 2 as well as Stage 3. The chain from principle [34] through to geometry dependence [40], field correlation with component durability [30], charge-to-depth conversion [41], and environmental parametrisation [42] makes this the best-validated sensing chain in the atmospheric literature—D2 evidence in the terms of Section 2.1—and the appropriate benchmark for any new material claiming aerospace relevance. Its limitation is that measured current depends on the electrode’s geometry, area ratio, electrolyte composition, and surface condition [40,42], so charge-to-depth conversion is environment-specific.

LPR and EIS apply a controlled perturbation and return corrosion rate estimates and interfacial mechanism information, respectively [28,29]. EIS resolves passive-film degradation, coating integrity, charge-transfer resistance, and diffusion, and is the standard tool for assessing the anodised and sol–gel systems airframe sensors must coexist with [61,66,84]. Both were developed for immersed cells and degrade in the thin, discontinuous, high-resistivity electrolytes typical of enclosed compartments; Tan’s treatment of measurement in highly resistive and inhomogeneous media [36] is the essential reference. Free-corrosion probes and zero-resistance ammetry (ZRA) use representative alloy electrodes, improving the material’s representativeness at the cost of demanding instrumentation [28,36].

Electrochemical noise records spontaneous fluctuations without external stimulation, its transients associated with pit initiation, and passive-film rupture [35]. It is, among the techniques reviewed here, the one whose primary evidence stage is most clearly 4, with localised propagation, which matters because that is what governs integrity in AA2024-T3 and AA7075-T6 [4,8,9]. Its weakness is interpretive: the physical meaning depends strongly on the signal processing applied, and non-stationarity complicates statistical treatment [35,36]. EN is high in information content and low in deployment readiness.

### 5.2. Electrical Transduction

ER probes measure the increase in resistance of a sacrificial track as its cross-section is consumed, giving a direct cumulative measure of Stage 5 [31,32,33]. Interpretation is simpler than for electrochemical methods because the output relates to geometry rather than the instantaneous reaction rate, and automated systems have demonstrated the sensitivity required for atmospheric rates: sputtered tracks of 50–800 nm resolve loss on an atomic scale [49], commercial loggers reach 1–10 nm depending on the track material and thickness with two-year autonomy [50], and sub-angstrom (<10^−10^ m) depth resolution has been reported under the VDA cyclic salt-spray/wet/dry/freeze test, though with pronounced hysteresis on carbon steel [51]. The response under multi-droplet wetting depends on the droplet geometry and wetting cycles, requiring high temporal resolution [32]. Shin et al. compared commercial ER and LPR devices against ultrasonic thickness measurement—one of few direct sensor versus independent method comparisons available [33]. The limitation is morphological: an ER track integrates loss along its length, so uniform thinning and a single deep pit of equal section loss read identically, and the resulting systematic under-reading under localised attack has now been quantified analytically and experimentally [91]. Given that attacks in aerospace aluminium proceed by grain boundary penetration and grain etch-out [4], this is a material shortcoming.

Passive interrogation of the same principle has been demonstrated with an RFID tag carrying a 30 nm Cu sensing layer, giving 0.4 dB nm^−1^ [92] and addressing the energy autonomy requirement of Section 8. Surface conductance sensors address Stage 2 reliably and cheaply but establish only that conditions permitting corrosion are present, not that corrosion occurred—a distinction often blurred when such devices are called corrosion sensors.

### 5.3. Optical and Photonic Transduction

Fibre-optic and colorimetric sensors respond to changes in the intensity, wavelength, fluorescence, absorption, or refractive index caused by corrosion-related chemistry [38,60]. They are immune to electromagnetic interference and are lightweight and multiplexable along a single fibre, suiting distributed monitoring. Venancio et al. developed optical sensors specifically for aluminium corrosion in airframes, and these remain one of very few examples designed for this application rather than adapted to it [38]. Fibre Bragg gratings are the mature platform and are already used for strain, temperature, and pressure [93]; corrosion sensing with them proceeds indirectly, through etched cladding, a sacrificial coating whose loss changes the strain state, or strain from the corrosion products [93,94]. The general limitation is that the detected quantity is a secondary indicator whose transport to the sensing layer is itself uncertain, with photochemical ageing and optical path contamination adding drift [38,60].

### 5.4. Electromagnetic Transduction

Eddy current testing, pulsed eddy current, and related methods respond to conductivity, permeability, and geometry, placing their primary evidence at Stages 5–6 [39,71,85,95]. They are the established means of detecting material loss around fasteners and in multilayer assemblies. Hernandez et al. applied PEC thermography to corrosion under insulation on painted aluminium aircraft structures [39]; classification of pulsed eddy current giant magnetoresistance (GMR) data on aircraft structures has been demonstrated [96]; frequency-spectrum and time–frequency analysis have resolved hidden defects and, specifically, hidden corrosion in multilayer aluminium assemblies (the lap-joint geometry) [97,98]; and magnetoresistive approaches address corrosion under insulation [99]. Eddy current scans of the USAF KC-135 fleet have been mined to link inspection data to corrosion damage, an early demonstration that inspection records carry recoverable degradation information [85]. Permanently installed arrays represent the convergence of this technique with the flexible electronics developments reviewed above; lift-off sensitivity, calibration stability, and installed durability remain the practical constraints.

### 5.5. Hybrid and Multifunctional Transduction

Combining mechanisms within one platform allows several cascade stages to be observed simultaneously [54,56], and independent indicators at Stages 2, 3, and 5 constrain each other’s interpretation in a way no single measurement can. The cost falls precisely on deployability: interfacial compatibility, cross-sensitivity between channels, calibration transfer, and reproducibility of a multi-material fabrication sequence. Hybrid platforms should be assessed in terms of manufacturing reproducibility alongside analytical performance.

Across the transduction mechanisms reviewed in this section, primary evidence stages and field validation maturity trade off in the same direction as the sensing material and representativeness (Section 4.9). ACM/galvanic and ER methods are the best field-validated (D2) but report early or averaged stages (2–3 and 5, respectively); electrochemical noise addresses Stage 4, the stage that governs integrity in AA2024-T3 and AA7075-T6, but remains interpretively immature; electromagnetic methods reach Stages 5–6 with spatial resolution only at the cost of requiring a discrete inspection event rather than continuous coverage. No mechanism reviewed here combines Stage 4/5 primary evidence with both continuous operation and field validation. Closing that combination, rather than improving any single mechanism’s sensitivity, is the practical target developed in Section 7.

## 6. From Material Performance to Engineering Relevance

**Figure 3:**
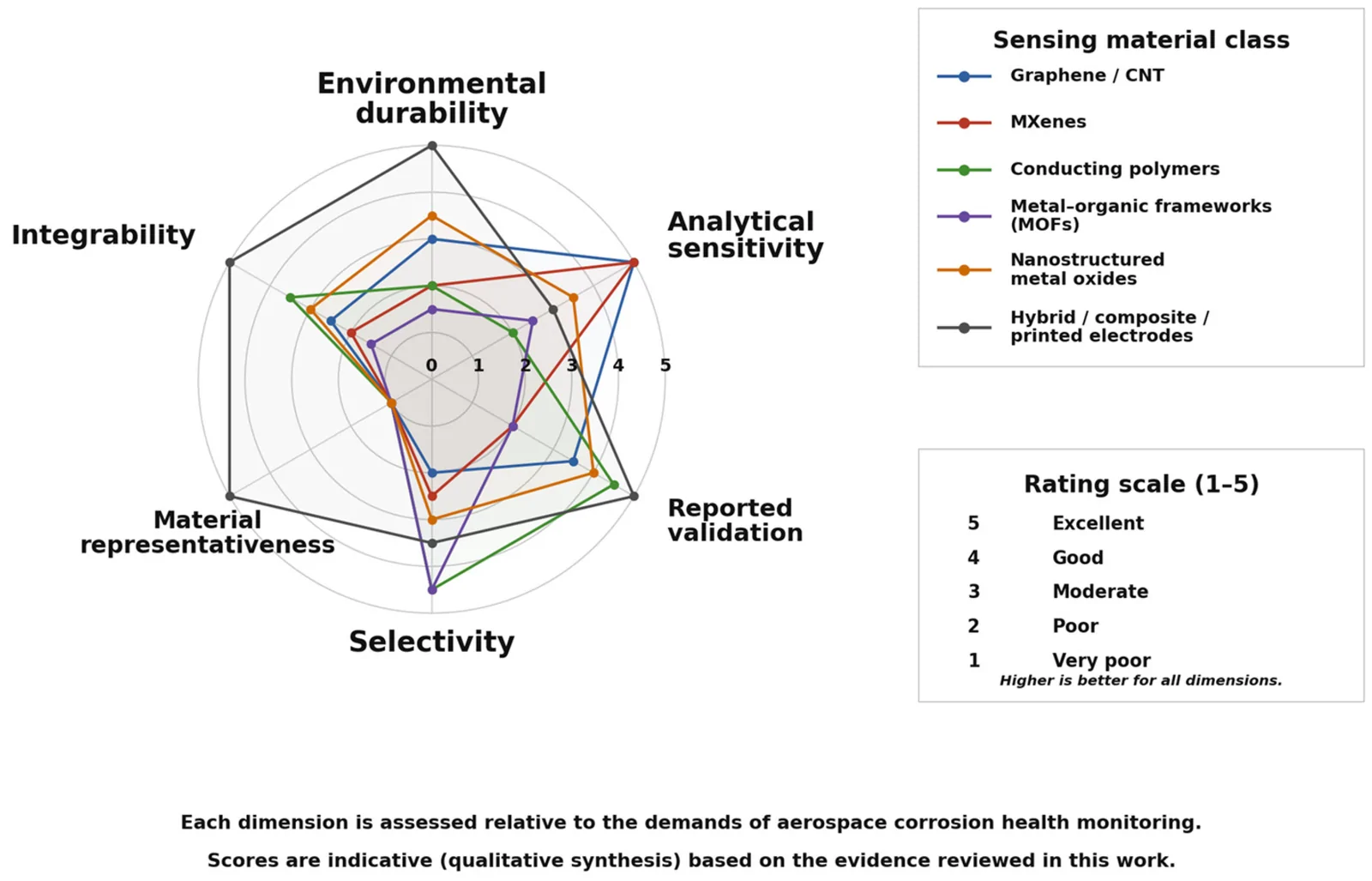
Qualitative assessment of sensing material classes against six criteria. Scores are ordinal, reflect the authors’ reading of the reviewed corpus (Section 2, Table 2), and are indicative rather than measured; no inter-rater comparison was performed. The scoring basis is as follows. Sensitivity: 5 = sub-ppm or equivalent, 1 = insufficient without amplification; selectivity: 5 = species-specific by design, 1 = responds to any conductivity change; environmental durability: 5 = stable over a maintenance interval under cyclic wetting, 1 = degrades within weeks; material representativeness: 5 = degrades via the same metallurgical mechanism as AA2024/AA7075, 1 = unrelated interface chemistry; integrability: 5 = conformable and deposited by an airframe-compatible process, 1 = requires laboratory instrumentation; reported validation level: 5 = in-service trial, 4 = field exposure, 3 = outdoor atmospheric, 2 = accelerated laboratory, 1 = single-condition laboratory. Each score carries an estimated uncertainty of ±1 scale point, given the absence of an inter-rater comparison; the figure should be read as a structured qualitative synthesis, not a measured comparison. The 1–5 rating scale shown in the figure corresponds to 1 = very poor, 2 = poor, 3 = moderate, 4 = good and 5 = excellent.

**Figure 4:**
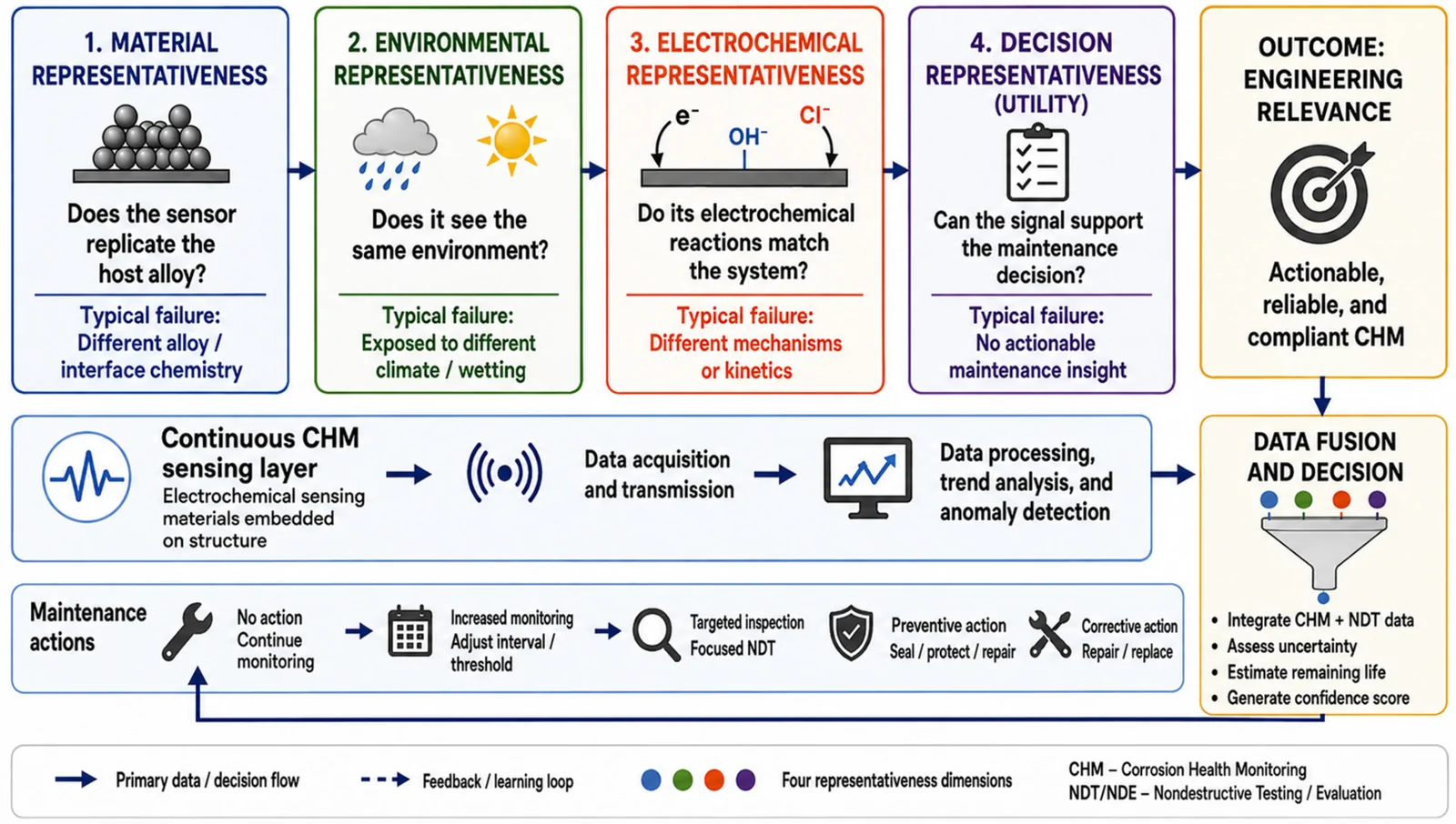
Engineering relevance framework. Each dimension is a necessary condition; failure at any gate prevents a sensor’s response from constituting engineering evidence about the monitored structure. The typical failure mode is given beneath each gate.

Section 4 and Section 5 establish what current sensing materials and mechanisms can detect, summarised qualitatively across the material classes in Figure 3. This section addresses the harder question of when that detection constitutes evidence about a structure. We propose an engineering relevance framework with four dimensions. Three are forms of representativeness in the strict sense—the degree to which the sensing element reproduces some property of the monitored system. The fourth, decision relevance, is different in kind: it concerns what the signal licenses rather than what it reproduces. It is retained in the framework because it is where the chain from measurement to action actually breaks. The framework (Figure 4) is a conceptual synthesis offered as a structured hypothesis, not an established model. Table 6 applies it to the principal sensor families.

### 6.1. Material Representativeness

Material representativeness is the extent to which the sensing element reproduces the degradation behaviour of the monitored alloy under identical exposure.

As set out in Section 1, the corrosion behaviour of AA2024-T3 and AA7075-T6 is governed by microstructural heterogeneity—intermetallic distribution, precipitate chemistry, grain boundary condition, and passive-film stability [2,3,4,5,6,7,8,9,10]. A sensing material designed for maximum charge-transfer efficiency has none of these features. Its response describes its own interface, and the inference related to the alloy passes through an empirical correlation that is valid only under the conditions in which it was established.

Sensing elements fabricated from the monitored alloy invert this trade-off. ER probes, sacrificial coupons, and electrochemical probes of representative composition degrade through the same metallurgical mechanisms as the structure [31,33], and ACM sensors using representative electrode pairs have shown a transferable correlation with component durability [30]. Their analytical sensitivity is lower than that of nanostructured layers; their engineering relevance is higher.

Modern airframes complicate this further. Aluminium alloys, titanium, stainless steel, carbon-fibre-reinforced polymers, adhesives, and multilayer coating systems coexist within a single joint, producing galvanic couples that no single sensing material reproduces. Representativeness is a property of the sensor–location pair, not of the sensor alone.

The productive response is hybridisation in a specific sense: pairing a representative metallic sensing element, which sets the degradation mechanism, with a high-performance transducer, which sets the signal’s quality. One concrete route is a coupon or thin foil of the actual alloy, with the correct temper, coupled to a high-performance transducer; a foil retains the microstructure that governs degradation, whereas an electrodeposited layer of matching composition does not. Where a deposited element is used for fabrication reasons, its response should be treated as compositionally representative and electrochemically calibrated rather than metallurgically identical to the structure.

Pairing a representative alloy element with a high-performance transducer, proposed above as the practical response to the sensitivity–representativeness trade-off, introduces its own interface. A bimetallic or metal/carbon interface will polarise galvanically if not electrically isolated; any gap or adhesive discontinuity at the interface can retain electrolyte and initiate a crevice attack; differential thermal expansion between a metallic foil and a polymer- or ceramic-based transducer will generate cyclic mechanical stress at the bond line over an airframe’s temperature range; and any encapsulation applied to protect the transducer (Section 6.2) must not itself seal the electrolyte against the alloy foil. These are the same categories of risk documented for CFRP/alloy joints in Section 4.8. No sensing material study reviewed here reports a hybrid platform tested against them. The available mitigations are correspondingly the same: electrical isolation layers of the kind evaluated for composite joints by Srinivasan et al. [75], mechanically compliant rather than rigid bonding, and encapsulation characterised explicitly as a transfer function (Section 6.2) rather than only as a barrier. Their application to a foil–transducer assembly, rather than to a structural joint, has not been demonstrated and is added as an experimental priority in Table 7.

**Table 6:** Engineering relevance assessment of the principal sensor families. Ratings are ordinal and reflect the authors’ synthesis of the reviewed corpus (Table 2, Section 6.1, Section 6.2, Section 6.3 and Section 6.4). High denotes a demonstrated mechanistic or field correspondence with the monitored alloy or process, Medium denotes a plausible but not directly tested correspondence, and Low denotes an acknowledged mismatch or absence of evidence. Ratings are independent of the six-criterion scoring used in Figure 3; they are indicative rather than measured, and no inter-rater comparison was performed. The final column identifies the dimension that limits engineering usefulness in each case.

Sensor Family	Material Repr.	Environmental Repr.	Electrochemical Repr.	Decision Relevance	Limiting Dimension	Refs.
Graphene/CNT electrodes	Low	Medium	Low–Medium	Low	Material	[17,21,22]
MXene-based devices	Low	Medium	Medium	Low	Measurement stability	[23,52,53,54]
Conducting polymer films	Low	Medium–High	Medium	Low	Measurement stability	[20,55]
MOF sensing layers	Low	Low–Medium	Low	Very low	Demonstrated corrosion relevance	[24,25,56,57,58,59]
Printed composite arrays	Low	High	Low–Medium	Low	Reproducibility	[19,22]
ACM/galvanic (representative pair)	Medium–High	High	Medium–High	Medium	Calibration transfer	[30,34,40,41,42]
ER probes (representative alloy)	High	High	Medium	Medium	Morphology averaging	[31,32,33]
Fibre-optic airframe sensors	Low	High	Medium	Low–Medium	Analyte transport	[38,60]
ET/PEC (installed or periodic)	High	n/a	n/a	High	Requires inspection event	[39,85]

**Table 7:** Current limitations, the experimental action required, the materials science perspective, and the expected engineering impact.

Current Limitation	Required Experimental Action	Materials Science Perspective	Expected Engineering Impact	Refs.
Sensitivity treated as the primary figure of merit	Report drift, hysteresis, recovery, and baseline stability over at least one scheduled maintenance interval of combined cyclic exposure	Characterise ageing of the active layer, not only its initial response	Selection of materials that survive a maintenance interval	[20,23,27,29]
Processing parameters reported without stability data	Parametric study of deposition variables against multi-month stability	Link electrode morphology to degradation of the sensing function	Reproducible, specifiable fabrication	[20,55,61,62]
Anodic layer used only for protection	Fill anodic pores with electroactive species; test against bare anodised reference	Porous Al_2_O_3_ as sensor substrate, isolation, and protection simultaneously	Sensing integrated into a qualified airframe process	[62]
Sensor-to-sensor variation unquantified	Report mean ± SD across a stated population of nominally identical devices	Process control as a materials variable	Comparable readings across an airframe and a fleet	[19,22]
Sensor’s response not compared with alloy’s behaviour	Parallel exposure of sensing element and alloy coupon; metallography of both	Match degradation mechanism, not only signal amplitude	Transferable correlations between environments	[2,3,4,5,9]
No standardised validation protocol	Staged protocol: laboratory, outdoor, demonstrator, in-service	Comparable exposure conditions across studies	Maturity that can be stated rather than claimed	[27,29]
Laboratory metrics only	Report false alarm rate, POD for defined corrosion states, threshold repeatability	Optimise specificity as well as sensitivity	Thresholds defensible in a maintenance context	[33,37]
ML trained on sensor data alone	Build datasets pairing sensor records with NDT-confirmed, metallographically verified damage	Ground truth as a materials characterisation problem	Models that predict damage rather than signal	[37,39,85,100]
Power and interrogation of embedded sensors	Demonstrate passive or energy-harvesting interrogation over a maintenance interval	Low-power transducers; printable antennas, and interconnectors	Deployment in inaccessible compartments	[17,19]

Figure 3 summarises this trade-off across the six classes reviewed in Section 4. Carbon nanomaterials and MXenes score highest on analytical sensitivity but lowest on material representativeness [17,21,22,23,52,53]; MOFs score highest on selectivity by design but lowest on demonstrated corrosion relevance [24,25,56,57,58,59]; conducting polymers and printed composites score highest on integrability, reflecting their airframe-compatible deposition routes, but lowest on long-term stability [19,20,22,55]; and representative alloy elements invert the pattern, scoring highest on material representativeness at the cost of sensitivity [30,31,33]. No class scores highly on more than two or three of the six criteria simultaneously.

### 6.2. Environmental Representativeness

Environmental representativeness is the degree to which the sensing element experiences the exposure that acts on the structure.

**Figure 5:**
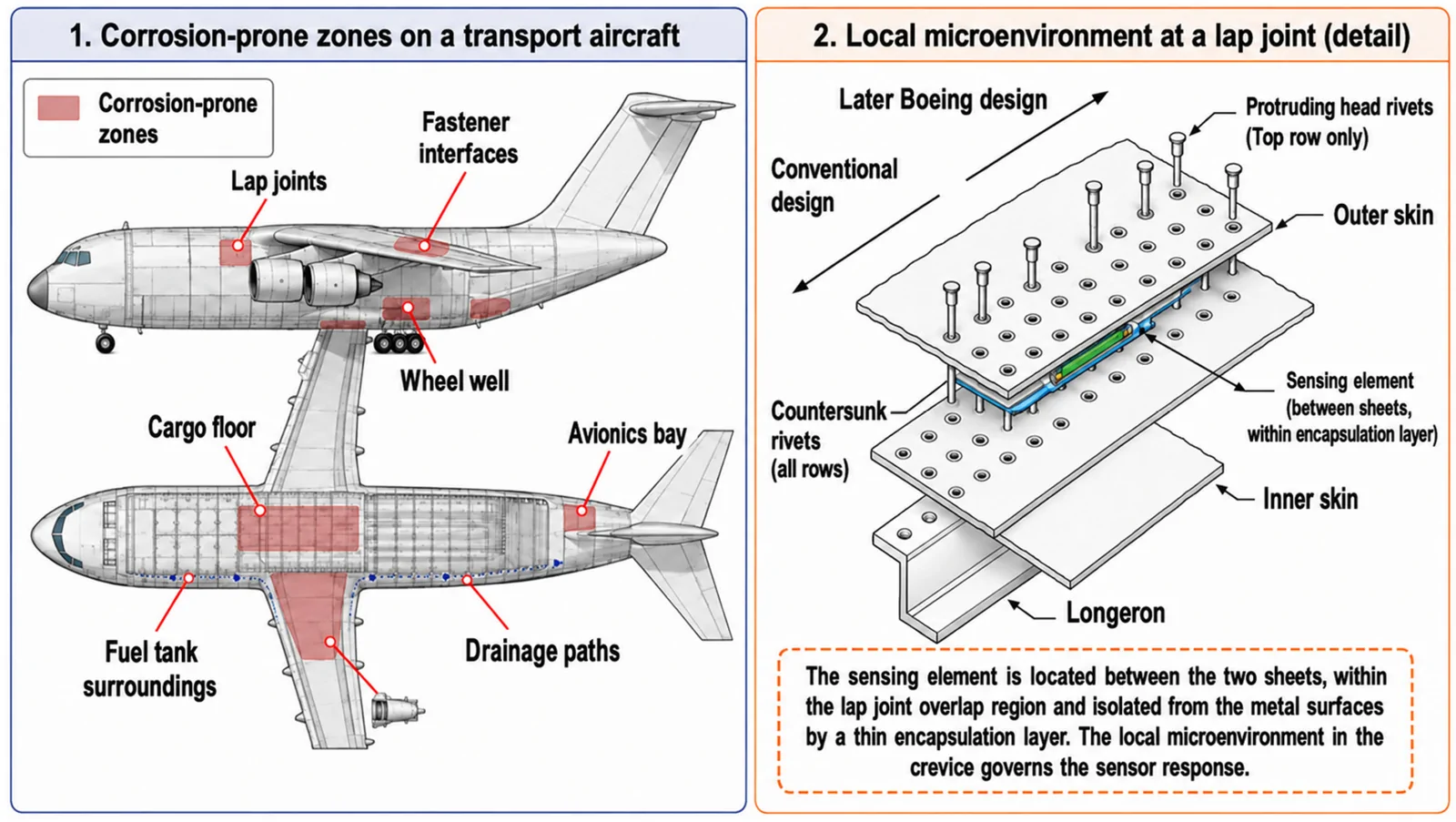
Corrosion-prone zones in a transport aircraft structure and the local microenvironment at a lap joint. The inset shows the encapsulation layer acting as a transfer function between the structural electrolyte film and the sensing element.

Atmospheric corrosion is controlled by the microenvironment. Humidity, chloride deposition, wet–dry cycling, electrolyte retention, and oxygen availability vary over millimetre distances [27,36], so two identical sensors in one compartment can report differently because one sits in a drainage path. Zhang et al. demonstrated this at the finest scale, with the response depending on individual droplets’ geometry and the wetting history [32], and correlation transfer between exposure environments requires explicit treatment [30,42]. In aircraft, corrosion concentrates in confined compartments, lap joints, fastener interfaces, drainage paths, cargo bays, and wheel wells (Figure 5), where electrolyte persists long after the external surfaces have dried; neither meteorological data nor a sensor mounted on an accessible external surface for convenience can represent these locations.

Miniaturisation has made placement inside these zones feasible, but placement alone is insufficient. The installation method, encapsulation, adhesive, sensor thermal mass and local geometry all modify the electrolyte film the sensor experiences relative to the film on the adjacent structure. An encapsulation that protects the sensing layer from contamination may also prevent it from wetting when the structure becomes wet. Encapsulation is a transfer function between the structure and sensor, and it should be characterised as one—reported and, where possible, measured against a co-located bare coupon.

Since the encapsulation chemistries available are the same hybrid sol–gel and conversion systems now being qualified as chromate replacements [61,63,65,66], their barrier properties are already documented; what is missing is their characterisation as a sensing transfer function rather than as a barrier.

### 6.3. Electrochemical Representativeness

Electrochemical representativeness is the capability of the sensing element to respond to the same electrochemical processes that govern initiation and propagation in the structural alloy.

This is distinct from material representativeness: a sensor may be made from the correct alloy and still fail if its electrode geometry, area ratio, or electrolyte access changes which reactions are rate-limiting [40,41,42]. Under thin, discontinuous films, the assumptions underlying LPR and EIS (uniform current distribution, defined electrolyte resistance) are not satisfied [36], so interpretations imported from immersed cell electrochemistry do not transfer. Localised corrosion is the specific difficulty. Integrity in aerospace aluminium is governed by pitting and intergranular attacks, which produce transient rather than steady signals [4,35], so area-averaged corrosion rates systematically under-represent the process that matters. EN addresses this directly but has not been reduced to a deployable indicator [35,36].

A sensing system is electrochemically representative when the reactions dominating its signal are the reactions dominating the alloy’s degradation at that location. Demonstrating this requires parallel exposure of the sensing element and the alloy coupon under identical conditions, with post-exposure metallography of both. Such comparisons are rare in the sensing material literature, and their absence is why most reported correlations cannot be transferred between environments.

### 6.4. Decision Relevance

Decision relevance is the extent to which a measured signal can be converted into a defensible engineering decision. Unlike the three representativeness dimensions, it is not a property of the sensor–structure correspondence but of the inference drawn from it, and it is separable into three distinct components that the literature often conflates: measurement stability (drift, hysteresis, recovery), uncertainty (the propagated budget below), and decision utility (whether the resulting evidence level supports the action taken).

Every conversion from signal to action requires answers to five questions: what physicochemical process generated the signal, which cascade stage it indicates, what uncertainty attaches to the measurement, whether the signal can be related to an inspection threshold, and what independent evidence is required before acting.

High sensitivity works against several of these. A nanostructured layer responding to small humidity or ion concentration changes will generate events that do not correspond to sustained corrosion. In a safety-critical maintenance context, the resulting false alarm rate is a first-order performance parameter, not a footnote. False positives cost inspection, downtime, and unnecessary disassembly; false negatives allow active corrosion to continue undetected. Their relative weight depends on the location’s criticality, accessibility, and failure consequence, so thresholds cannot be derived from laboratory sensitivity data alone.

Temporal interpretation matters as much as amplitude: a current transient may indicate breakdown that repassivates, whereas a slow resistance rise indicates continuing loss. Trend-based assessment is therefore more robust than threshold crossing, and its quality depends on baseline stability, drift, repeatability, hysteresis, and recovery, rather than on peak sensitivity. Uncertainty must also be propagated explicitly. Nine contributing terms are involved: material variability, fabrication tolerance, placement, encapsulation, cross-sensitivity, calibration drift, electronics, signal processing, and an imperfect sensor-to-structure relationship. Most published studies report only one of them.

**Figure 6:**
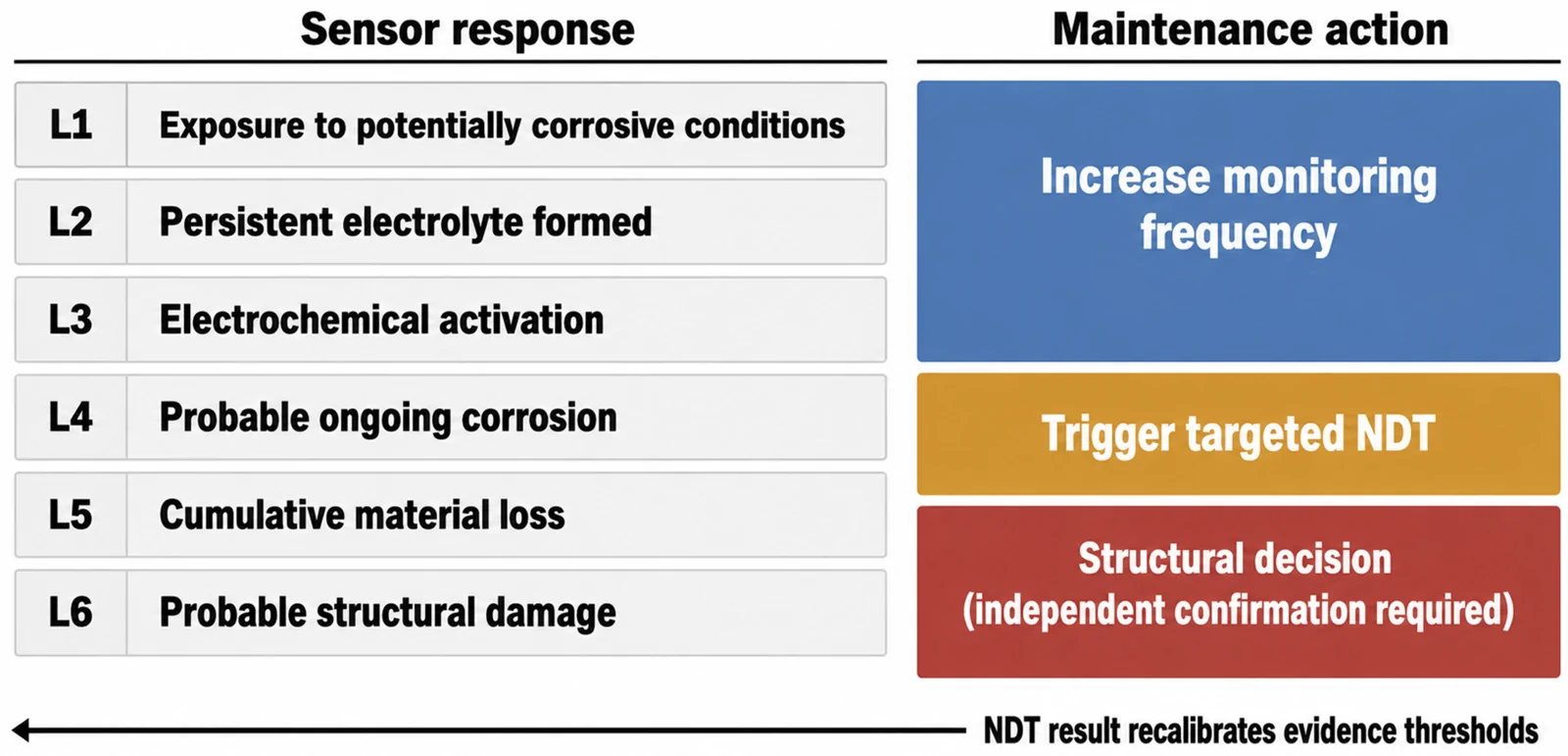
Staged evidence architecture linking a continuous sensor response to maintenance action. Evidence Levels 1–3 justify increased monitoring, Levels 4–5 trigger targeted nondestructive inspection, and only Level 6 with independent confirmation supports a structural decision. NDT results feed back to recalibrate the evidence thresholds. The scheme is a generic evidence architecture. The transition thresholds between levels are application-specific and are not fixed here, and a structural integrity assessment step sits between NDT and any structural disposition.

A monitoring system should therefore report a graded evidence level rather than a number against a limit (Figure 6) as follows:

1. Exposure to potentially corrosive conditions;
2. Formation of a persistent electrolyte;
3. Electrochemical activation;
4. Probable ongoing corrosion;
5. Cumulative material degradation;
6. Probable structural damage requiring confirmation.

This grading preserves the cascade distinction of Section 3 and prevents an environmental indicator from being used as though it were a damage measurement. It also makes the escalation logic explicit: Levels 1–3 justify increased monitoring, Levels 4–5 justify targeted NDT, and only Level 6 with independent confirmation justifies a structural decision.

Three distinct prediction targets are conflated in this literature and should be kept separate: prediction of the sensor’s own future signal, a time-series forecasting problem; prediction of a corrosion state, such as current density or depth, inferred from the signal through a calibration or model; and prediction of structural damage, such as crack initiation or section loss at a specific location, verified independently by NDT or metallography. Demonstrated predictive accuracy at the first level does not imply validity at the second or third; each requires its own ground truth. Machine learning fits naturally into Levels 1–4 and has demonstrated predictive capability for atmospheric corrosion sensor response at the first of these three levels [37]. It does not by itself raise the evidence level, because a model trained on sensor data predicts sensor data. Prediction accuracy and engineering reliability are separate quantities, and conflating them is the most common failure mode in the data-driven corrosion literature. Digital twin frameworks for condition monitoring [100] operate at a system level that presupposes the second or third target, but the ground truth against which such a framework was trained is rarely specified in this strand of the literature, which is the general constraint we highlight rather than a property of [100] specifically: the model is only as good as the ground truth behind it.

## 7. Engineering Validation Through Complementary Nondestructive Testing

The evidence grading of Section 6.4 leads to a conclusion that follows directly from the cascade model, with one important qualification. Environmental and electrochemical corrosion sensors, the classes reviewed in Section 4 and Section 5.1, Section 5.2 and Section 5.3, cannot finalise a structural integrity decision on their own because their primary evidence stages lie at 1–5 rather than 6. This does not apply to permanently installed electromagnetic arrays, whose primary evidence stage is 5–6 and which are better understood as automated NDT operating continuously than as corrosion sensors in the sense used here. The distinction matters for the system’s architecture: the two families are complementary, not interchangeable.

NDT supplies exactly what continuous sensing lacks, namely spatial resolution and a direct measurement of material loss and structural damage, and lacks exactly what continuous sensing supplies, namely temporal continuity. Eddy current and pulsed eddy current detect material loss and subsurface corrosion around fasteners and in multilayer assemblies; PEC thermography has been demonstrated on painted aluminium aircraft structure for corrosion under insulation, with its parameter limits established [39]. Eddy current scans of the USAF KC-135 fleet have been mined to establish empirical links between inspection data and corrosion damage—an early demonstration that inspection records themselves carry recoverable degradation information [85]. Ultrasonic thickness measurement quantifies section loss and has served as the reference method against which commercial corrosion sensors have been compared [33].

The productive architecture is staged rather than parallel. Continuous sensing identifies where and when the local environment became aggressive and whether electrochemical activation occurred; it ranks locations and triggers inspection. NDT determines whether damage of structural relevance is present. NDT supplies the location, size, depth, and extent of damage but does not by itself determine acceptability. The structural decision additionally requires the structural repair manual limits, residual strength and damage tolerance criteria, components’ criticality, and load history. The correct chain is therefore sensor → targeted NDT → structural integrity assessment → engineering disposition, with the sensing system determining the inspection interval and target—which is where the economic benefit lies.

This reframing also resolves the validation problem: a material validated only against a laboratory corrosion rate has been validated against a Stage 3 or Stage 5 proxy, whereas one validated against NDT-confirmed damage at the same location has been validated as an inspection-scheduling instrument, which is what it is. Shin et al. [33] and Venancio et al. [38] are among the few studies structured this way.

This is also where certification enters, and its absence from the sensing materials literature is conspicuous. Condition-based maintenance is not limited by transducers’ performance; the constraints repeatedly identified are the lack of standardised integration and certification requirements, the absence of agreed performance requirements for the sensing system, and the shortage of tools for converting monitoring output into maintenance decisions [101,102]. Guidance for fixed-wing aircraft has been developed to address exactly this [103]. The economic case is real but conditional, and it runs in the opposite direction to the one usually assumed. Permanently installed sensing adds mass, and that mass propagates through the aircraft by the snowball effect on maximum take-off weight. Cusati et al. framed the question as a breakeven rather than a saving. In their multidisciplinary analysis, the direct operating costs remain below those of the reference aircraft for sensor installations, adding up to approximately 4% to the maximum take-off weight [101]. A corrosion monitoring system must therefore justify itself inside that mass budget, which places a hard constraint on the sensor count, wiring and interrogation hardware that the sensing materials literature rarely acknowledges. That benefit is only realisable if the monitoring output is admissible as evidence—which returns the argument to decision representativeness (Section 6.4).

A corrosion monitoring system aiming at certification therefore needs to state, in advance, the damage state it detects, the probability of detection for that state, the false alarm rate, and the inspection action each evidence level triggers. None of the sensing material studies reviewed here reports that set. This is not a criticism of the materials work, which was not conducted for that purpose; it is an observation that the gap between a laboratory sensitivity figure and a certifiable inspection instrument is wider than the literature generally acknowledges.

Practically, this requires paired datasets that scarcely exist: co-located continuous sensor records and periodic NDT results over multiple maintenance intervals on real structure, with metallographic ground truth where components are eventually removed. Generating such datasets is demanding and time-consuming, and it is arguably the highest-value contribution now available to this field.

## 8. Research Challenges and Specific Experimental Priorities

The priorities below are stated as experiments rather than aspirations, on the grounds that “further research is needed” is not a research direction. Three deserve emphasis because they are experimentally tractable, clearly defined, and almost entirely absent from the literature.

The first is parallel exposure: simultaneous exposure of the sensing element and a coupon of the monitored alloy under identical conditions, with post-exposure metallography of both, characterised to the standard now routine in the AA2024 literature [2,3,4,5,9]. Without it, electrochemical representativeness cannot be established and the reported correlations cannot be transferred between environments.

The second is sensing functionality integrated into the anodic layer. Tartaric–sulphuric and phosphoric–sulphuric anodising are already qualified for airframes and produce a porous oxide that is a natural host for electroactive species [62,70,71]. Pore filling followed by exposure testing against a bare anodised reference is a well-defined experiment that has not been reported.

The third is processing–stability characterisation. For each route in Section 4.7, deposition parameters reported together with multi-month stability data. This requires parametric study of the processes already qualified for airframes rather than new chemistry [61,62,68,69,79], and it is the most tractable gap identified in this review.

The remaining priorities are set out in Table 7.

## 9. Conclusions

1. Corrosion monitoring signals are stage-specific in their primary evidence content. Environmental, electrochemical, cumulative, and structural measurements report different points in a six-stage degradation cascade, and a sensor cannot be validated against a quantity for which it is not the primary evidence. Many apparent disagreements about sensors’ accuracy in the reviewed corpus reduce to comparisons across different cascade stages.
2. Across the classes reviewed, high analytical sensitivity and high material representativeness appear difficult to obtain together—an apparent trade-off rather than a demonstrated correlation, given the qualitative and non-systematic basis of the assessment. The surface chemistry that makes carbon nanomaterials, MXenes, and MOFs excellent transducers is what makes them electrochemically unlike AA2024-T3 and AA7075-T6, whose degradation proceeds by intermetallic dealloying, trenching, and grain boundary penetration. The reviewed evidence suggests a structural trade-off rather than a temporary limitation, making hybrid architectures the practical response: a representative metallic element sets the degradation mechanism, and a high-performance transducer sets the signals’ quality.
3. Electrochemical processing route appears to limit deployability at least as much as material chemistry. Electrodeposition, electropolymerisation, anodic oxidation, and electrophoretic deposition each determine the electrode’s morphology, active area, and long-term stability, yet processing parameters have rarely been reported alongside multi-month stability data in the corpus reviewed. Closing this gap requires a parametric study of the processes already qualified for airframes rather than new materials.
4. In multi-material joints, the sensing problem inverts. CFRP acts as a large-area cathode driving dissolution of the coupled alloy, while the cathodic intermediates degrade the composite, so a sensor there observes two coupled processes with different thresholds. Surface treatment spans nearly the full range of achievable protection depending on the process parameters alone—a galvanic current reduction of approximately 90% for high-voltage plasma electrolytic oxidation against roughly 12% for a conventional anodic film. For sensing, it relocates the joint’s operating potential relative to the pitting threshold, so sensor baselines must be set for the treated condition.
5. Environmental representativeness is set by installation and encapsulation rather than by miniaturisation. Encapsulation acts as a transfer function between the structural microenvironment and the sensing layer and should be characterised as one, against co-located bare coupons. The hybrid sol–gel and conversion chemistries emerging as chromate replacements provide candidate encapsulants whose barrier properties have already been documented.
6. Decision relevance is the limiting dimension in the reviewed corpus, and it decomposes into measurement stability, uncertainty, and decision utility—three properties the literature routinely conflates. A graded six-level evidence scheme preserves the distinction between corrosion activity and corrosion damage and makes the escalation logic explicit.
7. Environmental and electrochemical corrosion sensors are complementary to NDT by construction; permanently installed electromagnetic arrays are better classified as automated NDT. Continuous sensing supplies temporal continuity and inspection targeting; eddy current, ultrasonic, and thermographic methods supply the spatial resolution and damage measurement on which structural decisions rest. The economic case for corrosion health monitoring lies in optimising the inspection intervals, not in replacing inspection.

The most valuable contribution now available to this field is not a more sensitive material but a dataset in which continuous sensor records, NDT results, and metallographic ground truth are paired on real structures over multiple maintenance intervals.

## Figures and Tables

**Table 4:** Transduction techniques mapped to cascade stages, with the state of field validation and the performance limitation under a thin or discontinuous electrolyte.

Technique	Measurand	Cascade Stage	Temporal Resolution	Thin/Discontinuous Electrolyte	Field Validation Evidence (D-Level, Table 5)	Refs.
ACM/galvanic	Galvanic current, integrated charge	2–3	Seconds–minutes	Good; requires bridging film	Automotive field exposure; environmental parametrisation (D2)	[30,34,40,42]
ZRA/free-corrosion probe	Coupling current between alloy electrodes	3	Seconds	Limited by current magnitude	Laboratory	[28,36]
LPR	Polarisation resistance → corrosion rate	3	Minutes	Poor; assumptions violated	Compared against UT thickness (D2)	[28,29,33]
EIS	Impedance spectrum → interfacial mechanism	2–3	Minutes–hours	Poor; high solution resistance	Laboratory; standard for coating assessment (D4)	[28,29,61,66]
Electrochemical noise	Spontaneous current/potential transients	4	Milliseconds–seconds	Usable; interpretation difficult	Laboratory	[35,36]
ER probe	Track resistance → section loss	5	Hours–days	Good	Model atmospheres; multi-droplet; UT comparison (D2)	[31,32,33]
Surface conductance	Film conductance, time of wetness	2	Seconds	Good	Field-deployed in atmospheric studies (not assigned; background methodology)	[27,36]
Fibre-optic/colorimetric	Optical property change from corrosion chemistry	2–4 (indirect)	Seconds–minutes	Depends on analyte transport	Airframe-specific development (D3, closest to D1 among sources reviewed)	[38,60]
ET/PEC/PEC thermography	Conductivity, permeability, geometry	5–6	Per inspection	Not applicable	Painted aluminium aircraft structure; fleet records (direct NDT evidence, outside the D1–D4 sensing hierarchy)	[39,85]
Ultrasonic thickness	Remaining wall thickness	5–6	Per inspection	Not applicable	Reference method for sensor validation (D2)	[33]

**Table 5:** Quantitative performance reported in selected primary studies, with the exposure under which it was obtained and the independent method used to verify it. The corpus is the narrative selection of Section 2 and is not exhaustive; entries were included only where a numerical value could be traced to the source. The final column gives the evidence level of Section 2.1. Note that no entry reaches D1—none of the traceable quantitative performance figures identified here was obtained on an aerospace alloy under airframe-representative exposure, which is itself a result of this review.

Study	Sensing Element	Measurand	Exposure	Reported Quantitative Performance	Duration	Independent Validation	Ev. (D-Level)
Kouřil et al. [49]	Sputtered Cu, Ag, Fe, Pb, bronze tracks, 50–800 nm	ΔR/R_0_ → thickness loss	Indoor atmospheres organic acid vapours	Corrosion loss resolved on an atomic scale; Cu and Ag respond to RH and T change within minutes	Laboratory	Corrosivity classification	D2
Prošek et al. [50]	Zn, Fe, Cu, Ni ER tracks, several thicknesses	ΔR/R_0_ → corrosion depth	Indoor and outdoor atmospheres	Sensitivity 1–10 nm depending on the track material and thickness; corrosivity change detected within hours to tens of minutes; 2-year autonomous logger life	Field, up to 2 years	Parallel atmospheric exposure	D2
Prošek et al. [31]	Carbon steel and Zn ER sensors	ΔR/R_0_ → corrosion depth	VDA cyclic test: salt spray, wet, dry, freeze	Sub-angstrom (<10^−10^ m) depth resolution; steel rate 0.2–0.6 µm h^−1^ outside the freezing phase; pronounced hysteresis on carbon steel	Full VDA cycle	Mass loss	D2
Reiser et al. [91]	ER sensor tracks, analytical model + experiment	ΔR/R_0_ under non-uniform attack	Modelled and experimental localised corrosion	Quantifies systematic under-reading of ER sensors when an attack is localised rather than uniform	Laboratory	Numerical model	D2
Ahn et al. [41]	Fe/Ag ACM galvanic sensor	Galvanic current, integrated charge → depth	Salt-spray accelerated test, carbon and weathering steel	Output thresholds: >10 µA for rainfall, >0.1 µA for dew; charge-to-depth relation calibrated against mass loss	Accelerated test	Mass-loss coupons	D2
Shin et al. [33]	Commercial ER and LPR probes	Wall thickness loss	Pilot-scale closed-loop pipe flow	ER-measured metal loss 0.254 mm against 0.25 mm actual; LPR unreliable above its corrosion rate threshold	Closed-loop trial	Ultrasonic thickness	D2
El Masri et al. [92]	RFID tag with 30 nm Cu sensing layer	RF signal attenuation → thickness loss	Indoor atmospheric exposure	Sensitivity 0.4 dB nm^−1^ on a 30 nm Cu layer	Laboratory	ER reference	D3
Liu et al. [67]	AA7075 with PEO layer, coupled to CFRP	Galvanic current density, galvanic potential	3.5 wt% NaCl, zero-resistance ammetry	Bare couple 58.2 µA cm^−2^ at −0.685 V; PEO-700 6.2 µA cm^−2^ at −0.36 V (vs. Ag/AgCl); ≈90% reduction against ≈12% for TFSAA at equal 10–11 µm thickness	Immersion trial	SEM, XRD, EIS	D4
Sloan and Talbot [43]	T-300/934 graphite/epoxy coupled to Mg	Mechanical and morphological degradation	Natural seawater, 140 days	≈30% loss of apparent shear strength; localised delamination ≈5 mm y^−1^	140 days	Mechanical testing	D2
Sloan and Talbot [44]	Graphite/epoxy under anodic polarisation	Composite damage threshold	Aqueous electrolyte	Measurable damage at current densities as low as 1 µA cm^−2^	Laboratory	Microscopy	D2

Of the traceable quantitative performance figures in Table 5, all reach D2; none reaches D1, consistent with the corpus-wide distribution in Section 2.1 (D1, 0%; D2, 17.5%; D3, 16.5%; D4, 36.9%; unassigned, 29.1% of 103 references).

## Data Availability

No new data were created or analyzed in this study. Data sharing is not applicable to this article.

## Abbreviations

ACM	Atmospheric corrosion monitor
CHM	Corrosion health monitoring
CNT	Carbon nanotube
EIS	Electrochemical impedance spectroscopy
EN	Electrochemical noise
EPD	Electrophoretic deposition
ER	Electrical resistance
ET	Eddy current testing
GMR	Giant magnetoresistance
GO	Graphene oxide
IGC	Intergranular corrosion
LIG	Laser-induced graphene
LPR	Linear polarization resistance
MOF	Metal–organic framework
NDT	Nondestructive testing
PAA	Phosphoric acid anodising
PANI	Polyaniline
PEC	Pulsed eddy current
PEDOT	Poly(3,4-ethylenedioxythiophene)
POD	Probability of detection
PPy	Polypyrrole
QCM	Quartz crystal microbalance
PSA	Phosphoric–sulphuric acid anodising
REACH	Registration, Evaluation, Authorisation and Restriction of Chemicals
rGO	Reduced graphene oxide
SAA	Sulphuric acid anodising
SHM	Structural health monitoring
TSA	Tartaric–sulphuric acid anodising
UT	Ultrasonic testing
ZRA	Zero-resistance ammetry
